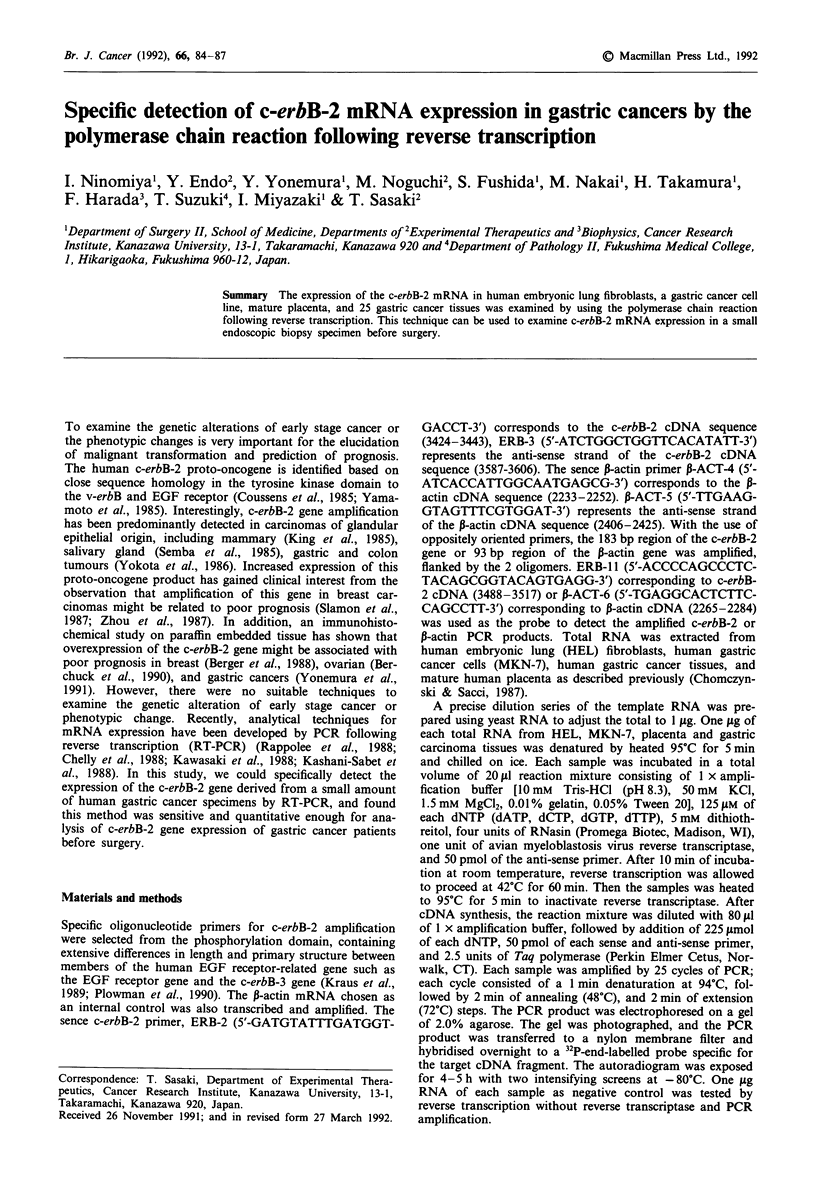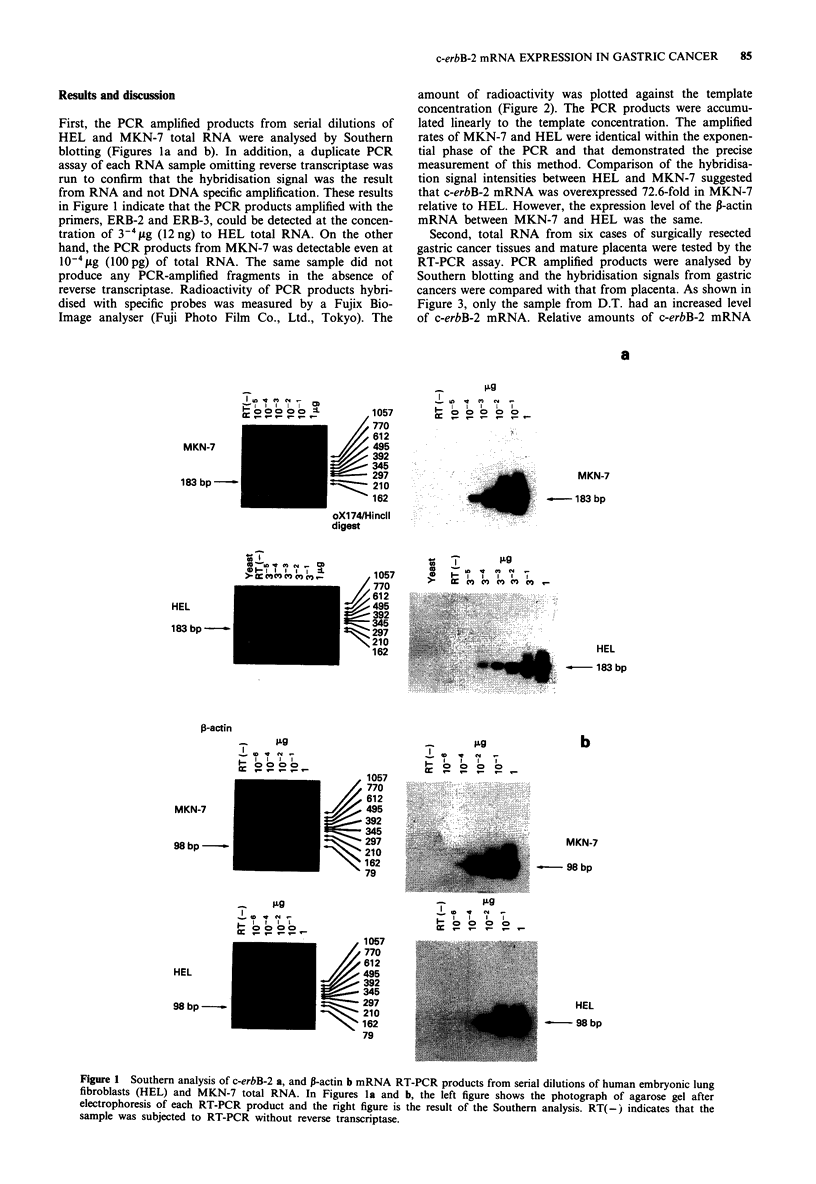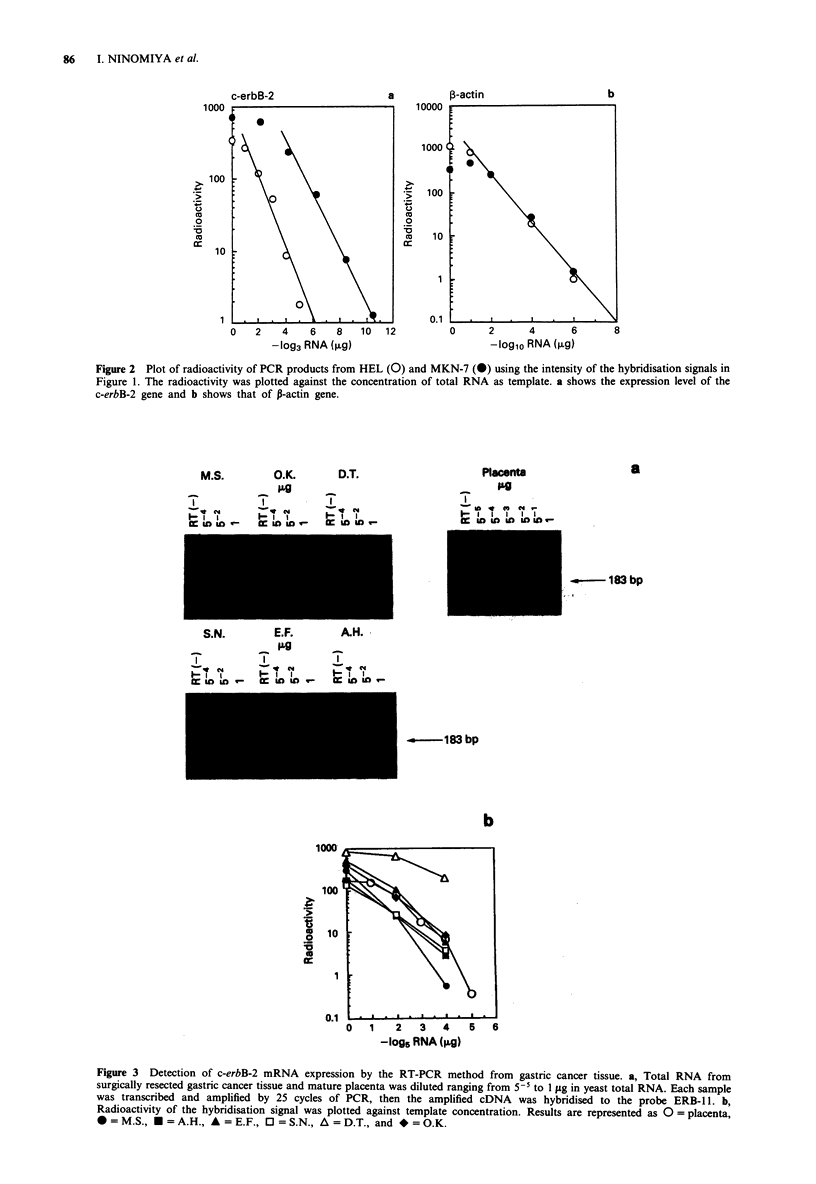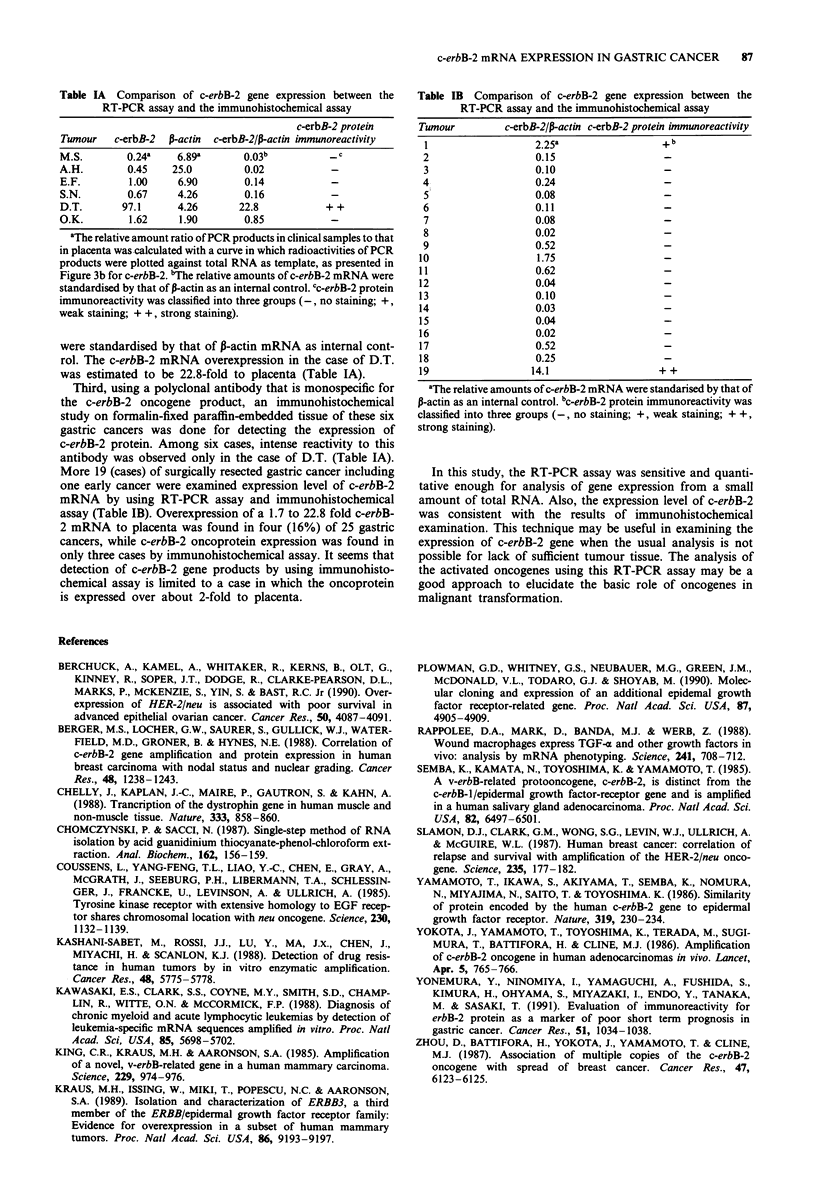# Specific detection of c-erbB-2 mRNA expression in gastric cancers by the polymerase chain reaction following reverse transcription.

**DOI:** 10.1038/bjc.1992.221

**Published:** 1992-07

**Authors:** I. Ninomiya, Y. Endo, Y. Yonemura, M. Noguchi, S. Fushida, M. Nakai, H. Takamura, F. Harada, T. Suzuki, I. Miyazaki

**Affiliations:** Department of Surgery II, School of Medicine, Kanazawa University, Japan.

## Abstract

**Images:**


Br. J. Cancer (1992), 66, 84–87 © Macmillan Press Ltd., 1992

### Specific detection of c-*erb*B-2 mRNA expression in gastric cancers by the polymerase chain reaction following reverse transcription

I. Ninomiya[1], Y. Endo[2], Y. Yonemura[1], M. Noguchi[2], S. Fushida[1], M. Nakai[1], H. Takamura[1], F. Harada[3], T. Suzuki[4], I. Miyazaki[1] & T. Sasaki[2]

[1]*Department of Surgery II, School of Medicine, Departments of* [2]*Experimental Therapeutics and* [3]*Biophysics, Cancer Research Institute, Kanazawa University, 13-1, Takaramachi, Kanazawa 920 and* [4]*Department of Pathology II, Fukushima Medical College, 1, Hikarigaoka, Fukushima 960-12, Japan.*

**Summary** The expression of the c-*erb*B-2 mRNA in human embryonic lung fibroblasts, a gastric cancer cell line, mature placenta, and 25 gastric cancer tissues was examined by using the polymerase chain reaction following reverse transcription. This technique can be used to examine c-*erb*B-2 mRNA expression in a small endoscopic biopsy specimen before surgery.

To examine the genetic alterations of early stage cancer or the phenotypic changes is very important for the elucidation of malignant transformation and prediction of prognosis. The human c-*erb*B-2 proto-oncogene is identified based on close sequence homology in the tyrosine kinase domain to the v-*erb*B and EGF receptor (Coussens *et al.*, 1985; Yamamoto *et al.*, 1985). Interestingly, c-*erb*B-2 gene amplification has been predominantly detected in carcinomas of glandular epithelial origin, including mammary (King *et al.*, 1985), salivary gland (Semba *et al.*, 1985), gastric and colon tumours (Yokota *et al.*, 1986). Increased expression of this proto-oncogene product has gained clinical interest from the observation that amplification of this gene in breast carcinomas might be related to poor prognosis (Slamon *et al.*, 1987; Zhou *et al.*, 1987). In addition, an immunohistochemical study on paraffin embedded tissue has shown that overexpression of the c-*erb*B-2 gene might be associated with poor prognosis in breast (Berger *et al.*, 1988), ovarian (Berchuck *et al.*, 1990), and gastric cancers (Yonemura *et al.*, 1991). However, there were no suitable techniques to examine the genetic alteration of early stage cancer or phenotypic change. Recently, analytical techniques for mRNA expression have been developed by PCR following reverse transcription (RT-PCR) (Rappolee *et al.*, 1988; Chelly *et al.*, 1988; Kawasaki *et al.*, 1988; Kashani-Sabet *et al.*, 1988). In this study, we could specifically detect the expression of the c-*erb*B-2 gene derived from a small amount of human gastric cancer specimens by RT-PCR, and found this method was sensitive and quantitative enough for analysis of c-*erb*B-2 gene expression of gastric cancer patients before surgery.

### Materials and methods

Specific oligonucleotide primers for c-*erb*B-2 amplification were selected from the phosphorylation domain, containing extensive differences in length and primary structure between members of the human EGF receptor-related gene such as the EGF receptor gene and the c-*erb*B-3 gene (Kraus *et al.*, 1989; Plowman *et al.*, 1990). The β-actin mRNA chosen as an internal control was also transcribed and amplified. The sence c-*erb*B-2 primer, ERB-2 (5′-GATGTATTTGATGGT-GACCT-3′) corresponds to the c-*erb*B-2 cDNA sequence (3424–3443), ERB-3 (5′-ATCTGGCTGGTTCACATATT-3′) represents the anti-sense strand of the c-*erb*B-2 cDNA sequence (3587-3606). The sence β-actin primer β-ACT-4 (5′-ATCACCATTGGCAATGAGCG-3′) corresponds to the β-actin cDNA sequence (2233–2252). β-ACT-5 (5′-TTGAAG-GTAGTTTCGTGGAT-3′) represents the anti-sense strand of the β-actin cDNA sequence (2406–2425). With the use of oppositely oriented primers, the 183 bp region of the c-*erb*B-2 gene or 93 bp region of the β-actin gene was amplified, flanked by the 2 oligomers. ERB-11 (5′-ACCCCAGCCCTC-TACAGCGGTACAGTGAGG-3′) corresponding to c-*erb*B-2 cDNA (3488–3517) or β-ACT-6 (5′-TGAGGCACTCTTC-CAGCCTT-3′) corresponding to β-actin cDNA (2265–2284) was used as the probe to detect the amplified c-*erb*B-2 or β-actin PCR products. Total RNA was extracted from human embryonic lung (HEL) fibroblasts, human gastric cancer cells (MKN-7), human gastric cancer tissues, and mature human placenta as described previously (Chomczynski & Sacci, 1987).

A precise dilution series of the template RNA was prepared using yeast RNA to adjust the total to 1 μg. One μg of each total RNA from HEL, MKN-7, placenta and gastric carcinoma tissues was denatured by heated 95°C for 5 min and chilled on ice. Each sample was incubated in a total volume of 20 μl reaction mixture consisting of 1 × amplification buffer [10 mM Tris-HCl (pH 8.3), 50 mM KCl, 1.5 mM $MgCl_2$, 0.01% gelatin, 0.05% Tween 20], 125 μM of each dNTP (dATP, dCTP, dGTP, dTTP), 5 mM dithiothreitol, four units of RNasin (Promega Biotec, Madison, WI), one unit of avian myeloblastosis virus reverse transcriptase, and 50 pmol of the anti-sense primer. After 10 min of incubation at room temperature, reverse transcription was allowed to proceed at 42°C for 60 min. Then the samples was heated to 95°C for 5 min to inactivate reverse transcriptase. After cDNA synthesis, the reaction mixture was diluted with 80 μl of 1 × amplification buffer, followed by addition of 225 μmol of each dNTP, 50 pmol of each sense and anti-sense primer, and 2.5 units of *Taq* polymerase (Perkin Elmer Cetus, Norwalk, CT). Each sample was amplified by 25 cycles of PCR; each cycle consisted of a 1 min denaturation at 94°C, followed by 2 min of annealing (48°C), and 2 min of extension (72°C) steps. The PCR product was electrophoresed on a gel of 2.0% agarose. The gel was photographed, and the PCR product was transferred to a nylon membrane filter and hybridised overnight to a $^{32}$P-end-labelled probe specific for the target cDNA fragment. The autoradiogram was exposed for 4–5 h with two intensifying screens at −80°C. One μg RNA of each sample as negative control was tested by reverse transcription without reverse transcriptase and PCR amplification.

Correspondence: T. Sasaki, Department of Experimental Therapeutics, Cancer Research Institute, Kanazawa University, 13-1, Takaramachi, Kanazawa 920, Japan.
Received 26 November 1991; and in revised form 27 March 1992.

**Results and discussion**

First, the PCR amplified products from serial dilutions of HEL and MKN-7 total RNA were analysed by Southern blotting (Figures 1a and b). In addition, a duplicate PCR assay of each RNA sample omitting reverse transcriptase was run to confirm that the hybridisation signal was the result from RNA and not DNA specific amplification. These results in Figure 1 indicate that the PCR products amplified with the primers, ERB-2 and ERB-3, could be detected at the concentration of $3^{-4}$ μg (12 ng) to HEL total RNA. On the other hand, the PCR products from MKN-7 was detectable even at $10^{-4}$ μg (100 pg) of total RNA. The same sample did not produce any PCR-amplified fragments in the absence of reverse transcriptase. Radioactivity of PCR products hybridised with specific probes was measured by a Fujix Bio-Image analyser (Fuji Photo Film Co., Ltd., Tokyo). The amount of radioactivity was plotted against the template concentration (Figure 2). The PCR products were accumulated linearly to the template concentration. The amplified rates of MKN-7 and HEL were identical within the exponential phase of the PCR and that demonstrated the precise measurement of this method. Comparison of the hybridisation signal intensities between HEL and MKN-7 suggested that c-*erb*B-2 mRNA was overexpressed 72.6-fold in MKN-7 relative to HEL. However, the expression level of the β-actin mRNA between MKN-7 and HEL was the same.

Second, total RNA from six cases of surgically resected gastric cancer tissues and mature placenta were tested by the RT-PCR assay. PCR amplified products were analysed by Southern blotting and the hybridisation signals from gastric cancers were compared with that from placenta. As shown in Figure 3, only the sample from D.T. had an increased level of c-*erb*B-2 mRNA. Relative amounts of c-*erb*B-2 mRNA

**Figure 1** Southern analysis of c-*erb*B-2 **a**, and β-actin **b** mRNA RT-PCR products from serial dilutions of human embryonic lung fibroblasts (HEL) and MKN-7 total RNA. In Figures **1a** and **b**, the left figure shows the photograph of agarose gel after electrophoresis of each RT-PCR product and the right figure is the result of the Southern analysis. RT(−) indicates that the sample was subjected to RT-PCR without reverse transcriptase.

**Figure 2** Plot of radioactivity of PCR products from HEL (○) and MKN-7 (●) using the intensity of the hybridisation signals in Figure 1. The radioactivity was plotted against the concentration of total RNA as template. **a** shows the expression level of the c-*erb*B-2 gene and **b** shows that of β-actin gene.

**Figure 3** Detection of c-*erb*B-2 mRNA expression by the RT-PCR method from gastric cancer tissue. **a**, Total RNA from surgically resected gastric cancer tissue and mature placenta was diluted ranging from $5^{-5}$ to 1 μg in yeast total RNA. Each sample was transcribed and amplified by 25 cycles of PCR, then the amplified cDNA was hybridised to the probe ERB-11. **b**, Radioactivity of the hybridisation signal was plotted against template concentration. Results are represented as ○ = placenta, ● = M.S., ■ = A.H., ▲ = E.F., □ = S.N., △ = D.T., and ◆ = O.K.

**Table IA** Comparison of c-*erb*B-2 gene expression between the RT-PCR assay and the immunohistochemical assay

| Tumour | c-erb*B-2* | *β-actin* | c-erb*B-2/β-actin* | c-erb*B-2 protein immunoreactivity* |
|---|---|---|---|---|
| M.S. | 0.24[a] | 6.89[a] | 0.03[b] | –[c] |
| A.H. | 0.45 | 25.0 | 0.02 | – |
| E.F. | 1.00 | 6.90 | 0.14 | – |
| S.N. | 0.67 | 4.26 | 0.16 | – |
| D.T. | 97.1 | 4.26 | 22.8 | ++ |
| O.K. | 1.62 | 1.90 | 0.85 | – |

[a]The relative amount ratio of PCR products in clinical samples to that in placenta was calculated with a curve in which radioactivities of PCR products were plotted against total RNA as template, as presented in Figure 3b for c-*erb*B-2. [b]The relative amounts of c-*erb*B-2 mRNA were standardised by that of β-actin as an internal control. [c]c-*erb*B-2 protein immunoreactivity was classified into three groups (–, no staining; +, weak staining; ++, strong staining).

were standardised by that of β-actin mRNA as internal control. The c-*erb*B-2 mRNA overexpression in the case of D.T. was estimated to be 22.8-fold to placenta (Table IA).

Third, using a polyclonal antibody that is monospecific for the c-*erb*B-2 oncogene product, an immunohistochemical study on formalin-fixed paraffin-embedded tissue of these six gastric cancers was done for detecting the expression of c-*erb*B-2 protein. Among six cases, intense reactivity to this antibody was observed only in the case of D.T. (Table IA). More 19 (cases) of surgically resected gastric cancer including one early cancer were examined expression level of c-*erb*B-2 mRNA by using RT-PCR assay and immunohistochemical assay (Table IB). Overexpression of a 1.7 to 22.8 fold c-*erb*B-2 mRNA to placenta was found in four (16%) of 25 gastric cancers, while c-*erb*B-2 oncoprotein expression was found in only three cases by immunohistochemical assay. It seems that detection of c-*erb*B-2 gene products by using immunohistochemical assay is limited to a case in which the oncoprotein is expressed over about 2-fold to placenta.

**Table IB** Comparison of c-*erb*B-2 gene expression between the RT-PCR assay and the immunohistochemical assay

| *Tumour* | c-erb*B-2/β-actin* | c-erb*B-2 protein immunoreactivity* |
|---|---|---|
| 1 | 2.25[a] | +[b] |
| 2 | 0.15 | – |
| 3 | 0.10 | – |
| 4 | 0.24 | – |
| 5 | 0.08 | – |
| 6 | 0.11 | – |
| 7 | 0.08 | – |
| 8 | 0.02 | – |
| 9 | 0.52 | – |
| 10 | 1.75 | – |
| 11 | 0.62 | – |
| 12 | 0.04 | – |
| 13 | 0.10 | – |
| 14 | 0.03 | – |
| 15 | 0.04 | – |
| 16 | 0.02 | – |
| 17 | 0.52 | – |
| 18 | 0.25 | – |
| 19 | 14.1 | ++ |

[a]The relative amounts of c-*erb*B-2 mRNA were standarised by that of β-actin as an internal control. [b]c-*erb*B-2 protein immunoreactivity was classified into three groups (–, no staining; +, weak staining; ++, strong staining).

In this study, the RT-PCR assay was sensitive and quantitative enough for analysis of gene expression from a small amount of total RNA. Also, the expression level of c-*erb*B-2 was consistent with the results of immunohistochemical examination. This technique may be useful in examining the expression of c-*erb*B-2 gene when the usual analysis is not possible for lack of sufficient tumour tissue. The analysis of the activated oncogenes using this RT-PCR assay may be a good approach to elucidate the basic role of oncogenes in malignant transformation.

### References

BERCHUCK, A., KAMEL, A., WHITAKER, R., KERNS, B., OLT, G., KINNEY, R., SOPER, J.T., DODGE, R., CLARKE-PEARSON, D.L., MARKS, P., MCKENZIE, S., YIN, S. & BAST, R.C. Jr (1990). Overexpression of *HER-2/neu* is associated with poor survival in advanced epithelial ovarian cancer. *Cancer Res.*, **50**, 4087–4091.

BERGER, M.S., LOCHER, G.W., SAURER, S., GULLICK, W.J., WATERFIELD, M.D., GRONER, B. & HYNES, N.E. (1988). Correlation of c-*erb*B-2 gene amplification and protein expression in human breast carcinoma with nodal status and nuclear grading. *Cancer Res.*, **48**, 1238–1243.

CHELLY, J., KAPLAN, J.-C., MAIRE, P., GAUTRON, S. & KAHN, A. (1988). Trancription of the dystrophin gene in human muscle and non-muscle tissue. *Nature*, **333**, 858–860.

CHOMCZYNSKI, P. & SACCI, N. (1987). Single-step method of RNA isolation by acid guanidinium thiocyanate-phenol-chloroform extraction. *Anal. Biochem.*, **162**, 156–159.

COUSSENS, L., YANG-FENG, T.L., LIAO, Y.-C., CHEN, E., GRAY, A., MCGRATH, J., SEEBURG, P.H., LIBERMANN, T.A., SCHLESSINGER, J., FRANCKE, U., LEVINSON, A. & ULLRICH, A. (1985). Tyrosine kinase receptor with extensive homology to EGF receptor shares chromosomal location with *neu* oncogene. *Science*, **230**, 1132–1139.

KASHANI-SABET, M., ROSSI, J.J., LU, Y., MA, J.X., CHEN, J., MIYACHI, H. & SCANLON, K.J. (1988). Detection of drug resistance in human tumors by in vitro enzymatic amplification. *Cancer Res.*, **48**, 5775–5778.

KAWASAKI, E.S., CLARK, S.S., COYNE, M.Y., SMITH, S.D., CHAMPLIN, R., WITTE, O.N. & MCCORMICK, F.P. (1988). Diagnosis of chronic myeloid and acute lymphocytic leukemias by detection of leukemia-specific mRNA sequences amplified *in vitro*. *Proc. Natl Acad. Sci, USA*, **85**, 5698–5702.

KING, C.R., KRAUS, M.H. & AARONSON, S.A. (1985). Amplification of a novel, v-*erb*B-related gene in a human mammary carcinoma. *Science*, **229**, 974–976.

KRAUS, M.H., ISSING, W., MIKI, T., POPESCU, N.C. & AARONSON, S.A. (1989). Isolation and characterization of *ERBB3*, a third member of the *ERBB*/epidermal growth factor receptor family: Evidence for overexpression in a subset of human mammary tumors. *Proc. Natl Acad. Sci. USA*, **86**, 9193–9197.

PLOWMAN, G.D., WHITNEY, G.S., NEUBAUER, M.G., GREEN, J.M., MCDONALD, V.L., TODARO, G.J. & SHOYAB, M. (1990). Molecular cloning and expression of an additional epidemal growth factor receptor-related gene. *Proc. Natl Acad. Sci. USA*, **87**, 4905–4909.

RAPPOLEE, D.A., MARK, D., BANDA, M.J. & WERB, Z. (1988). Wound macrophages express TGF-α and other growth factors in vivo: analysis by mRNA phenotyping. *Science*, **241**, 708–712.

SEMBA, K., KAMATA, N., TOYOSHIMA, K. & YAMAMOTO, T. (1985). A v-*erb*B-related protooncogene, c-*erb*B-2, is distinct from the c-*erb*B-1/epidermal growth factor-receptor gene and is amplified in a human salivary gland adenocarcinoma. *Proc. Natl Acad. Sci. USA*, **82**, 6497–6501.

SLAMON, D.J., CLARK, G.M., WONG, S.G., LEVIN, W.J., ULLRICH, A. & MCGUIRE, W.L. (1987). Human breast cancer: correlation of relapse and survival with amplification of the HER-2/*neu* oncogene. *Science*, **235**, 177–182.

YAMAMOTO, T., IKAWA, S., AKIYAMA, T., SEMBA, K., NOMURA, N., MIYAJIMA, N., SAITO, T. & TOYOSHIMA. K. (1986). Similarity of protein encoded by the human c-*erb*B-2 gene to epidermal growth factor receptor. *Nature*, **319**, 230–234.

YOKOTA, J., YAMAMOTO, T., TOYOSHIMA, K., TERADA, M., SUGIMURA, T., BATTIFORA, H. & CLINE, M.J. (1986). Amplification of c-*erb*B-2 oncogene in human adenocarcinomas *in vivo*. *Lancet*, **Apr. 5**, 765–766.

YONEMURA, Y., NINOMIYA, I., YAMAGUCHI, A., FUSHIDA, S., KIMURA, H., OHYAMA, S., MIYAZAKI, I., ENDO, Y., TANAKA, M. & SASAKI, T. (1991). Evaluation of immunoreactivity for *erb*B-2 protein as a marker of poor short term prognosis in gastric cancer. *Cancer Res.*, **51**, 1034–1038.

ZHOU, D., BATTIFORA, H., YOKOTA, J., YAMAMOTO, T. & CLINE, M.J. (1987). Association of multiple copies of the c-*erb*B-2 oncogene with spread of breast cancer. *Cancer Res.*, **47**, 6123–6125.